# The hypothalamic effects of PACAP on the hypothalamic-pituitary-gonadal axis in male mice

**DOI:** 10.3389/fendo.2025.1677085

**Published:** 2025-11-19

**Authors:** Péter Faludi, Klaudia Barabás, Ferenc Lengyel, Ildikó Udvarácz, Dániel Pham, Olivér Kisjós, Zsuzsanna Nagy, Dóra Reglődi, Gergely Kovács

**Affiliations:** 1 Institute of Physiology, Medical School, University of Pécs, Pécs, Hungary; 2 Department of Anatomy, HUN-REN-PTE PACAP Research Team, Medical School, University of Pécs, Pécs, Hungary

**Keywords:** PACAP, GnRH, kisspeptin, estrogen receptor alpha, androgen receptor

## Abstract

Pituitary adenylate cyclase-activating polypeptide (PACAP) is a member of the vasoactive intestinal peptide (VIP) neuropeptide family and plays a role in the regulation of several releasing hormones and tropic hormones. The hypothalamic-pituitary-gonadal (HPG) axis governs the synthesis and the release of sex hormones and the gametogenesis in all mammals. While the effects of PACAP on fertility is well-documented in females, much less data are available in males. The aim of our study was to examine potential structural and expressional changes in the hypothalamus that might underlie the fertility deficits observed in male PACAP knockout (KO) mice. To this end, we performed immunofluorescent, immunohistochemical and RNAscope *in situ* hybridization stainings to detect the protein and/or mRNA expression of gonadotropin-releasing hormone (GnRH), kisspeptin, estrogen receptor alpha (ERα) and androgen receptor (AR) in the hypothalamus. Our results revealed that the number and immunoreactivity of GnRH neurons were lower in the medial preoptic area (MPOA) in PACAP KO mice. In contrast, the number of kisspeptin neurons was higher in the rostral periventricular region of the third ventricle (RP3V) and the mid arcuate nucleus (ARC). Furthermore, higher number of *Esr1-*positive cells was found in the kisspeptin-rich RP3V and the ARC. Notably, less AR-positive cells, and more ERα-positive cells were detected in the MPOA demonstrating a possible misbalance between estrogenic and androgenic signaling. Our results suggest that neuroendocrine changes induced by PACAP deficiency in the hypothalamus might contribute to the development of reproductive dysfunction in PACAP-deficient males by disrupting normal HPG axis function.

## 1 Introduction

In the human body, gametogenesis and the synthesis of sexual steroids are regulated by the hypothalamic-pituitary-gonadal (HPG) axis (1). The hypothalamic GnRH neurons are key players in the HPG axis as they are responsible for the production of sexual steroids in both sexes (2). Pulsatile GnRH secretion is essential for the proper functioning of the HPG axis. The activity of GnRH neurons is modulated by several neural inputs of which kisspeptin neurons are the most remarkable ones. The main sexual steroids, namely estrogen, progesterone and testosterone in female and male mammals exert their negative or positive feedback effects on GnRH neurons via modulating the activity of kisspeptin neurons which, in rodents, are located in the arcuate nucleus (ARC) and the rostral periventricular region of the third ventricle (RP3V) (3, 4). The kisspeptin neuron population in the ARC serves as the “GnRH pulse generator” in both sexes, while the other kisspeptin group in the RP3V functions as the “GnRH surge generator” in females. In males, however, the role of the relatively small population of RP3V kisspeptin neurons is not yet known (3, 5). Sexual steroids are synthesized mainly in the gonads, but other organs such as brain, skin or adipose tissue also produce them changing the local hormonal levels (6). The blood levels of sexual steroids are affected by several factors such as age, sex, lifestyle and genetics (7).

The pituitary adenylate cyclase-activating peptide (PACAP) is a neuropeptide that is produced in many organs including the brain and the gonads. PACAP is a member of the vasoactive intestinal peptide (VIP)/glucagon/secretin family (8). PACAP was first identified in the late 1980s, when the 38-amino acid form (PACAP38) was isolated from ovine hypothalamic extracts (9). Subsequently, a shorter isoform comprising 27 amino acids, PACAP27, was identified the following year (10). PACAP has three receptors: the PACAP-specific PAC1-receptor (PAC1R) and the VPAC1- and VPAC2-receptor, which have similar affinity to PACAP and VIP (11). Both PACAP and its receptors are expressed in the hypothalamus, pituitary gland and in a variety of cell types in the gonads (12–14). PACAP-containing neurons and PACAP-immunoreactive fibers were found to be present throughout the hypothalamus in several species (15–18).

The effects of PACAP on the reproductive functions are well-studied in females, while much less is known in males (19–21). In female mice PACAP neurons in the ventral premamillary nucleus (PMV) of the hypothalamus monosynaptically contact a subset of ARC and RP3V kisspeptin neurons (22). Targeted deletion of PACAP from the PMV led to delayed puberty onset and impaired reproductive function in female, but not in male mice (22). In addition, mRNA of PAC1 receptors was detected in kisspeptin neurons from the ARC of female mice (23).

Whole-body PACAP KO females show fewer *Kiss1* mRNA-positive cells in ARC but not in RP3V (24). In contrast, no *in vivo* studies reported that genetic ablation of PACAP or blocking PACAP signaling modulates kisspeptin expression or the number of kisspeptin neurons in male mice. In an *in vitro* study, PACAP treatment resulted in an upregulation of kisspeptin mRNA expression in two hypothalamic cell models derived from male mice that express kisspeptin (25).

The effect of PACAP on GnRH mRNA expression was shown to be dependent on the route of the administration *in vivo*: intracerebroventricular injection stimulated expression, whereas intravenous administration produced the opposite effect (26). In male rats it was demonstrated by several studies that PACAP stimulates luteinizing hormone (LH) secretion *in vivo* (27, 28). *In vitro* studies also provided evidence that PACAP stimulates LH release in rats (29–33).

It was also demonstrated that PACAP stimulated testosterone secretion in primary Leydig cells and in immortalized cell lines (34, 35). In PACAP KO male mice with C57Bl/6 genetic background testicular aging was delayed, testosterone blood level was lower in 4-month-old animals with no change in LH or follicle-stimulating hormone (FSH) concentration. In addition, the amount of enzymes involved in the synthesis of testosterone was reduced in PACAP KO animals (36). Interestingly, in male mice with targeted pituitary overexpression of PACAP, testosterone blood level was also lower compared to wild-type animals. In addition, suppressed circulating LH and FSH levels with delayed timing of puberty were observed in these male animals (37).

Gametogenesis is also affected by PACAP. The diameter of sperm heads was found to be smaller in PACAP KO animals with Crlj: CD1 genetic background (38). The motility of mature sperm was reduced by PACAP_7–27_ hybrid antagonist in golden hamster (39). The regulation of the synthesis of a variety of proteins in rat spermatids and spermatocytes by PACAP was demonstrated *in vitro* (40). Finally, robust changes in the expression of several factors playing important roles in spermatogenesis were reported in PACAP KO animals (41).

All these data support that PACAP is a crucial element of the regulatory machinery of testosterone synthesis and spermatogenesis acting at all three levels (hypothalamic, pituitary, and gonadal) of the HPG axis.

To address the current lack of knowledge on how PACAP influences GnRH and kisspeptin neurons in males *in vivo*, this study aimed to evaluate the integrity of the hypothalamic kisspeptin–GnRH neural network and to examine androgen receptor (AR) and estrogen receptor alpha (ERα) expression in specific hypothalamic regions of PACAP KO male mice.

## 2 Materials and methods

### 2.1 Animals

12- to 14-weeks-old male homozygous PACAP KO (genetic background of Institute of Cancer Research (CD-1)) (42) and wild-type mice were used in all experiments (n=6–10 in both groups). Mice were kept under a 12:12 h light/dark cycle in the Animal House of the Department of Anatomy at the University of Pécs, according to the regulations of the European Community Council Directive and the Animal Welfare Committee of the University of Pécs. Animals were fed with regular chow and had access to water *ad libitum*. This animal study was approved by the Local Animal Care Committee of the University of Pécs (BA02/2000-24/2011 University of Pécs, Hungary).

### 2.2 Perfusion fixation and brain sectioning

To obtain reliable and comparable serum testosterone values we sacrificed the animals between 8:00 and 11:00 AM. 0.3-0.35 ml of 2.5% 2,2,2-tribromoethanol (Avertin, i.p.; Sigma, St Louis, MO, USA) was used to deeply anaesthetize the animals prior to perfusion. Prior to transcardial perfusion of the animals, body weight and the weights of both testes were measured. One testis was then immediately stored at -80 for subsequent analyses.

To wash blood out animals were transcardially perfused with 4–5 ml ice-cold phosphate buffered saline (PBS). Thereafter, tissues were fixed by perfusing 4% paraformaldehyde in PBS buffer for 30 minutes at room temperature (20 ml, pH 7.6). Perfusion-fixed brain samples were collected from the skull, postfixed at 4°C for overnight. Fixed brains were cryoprotected in 30% sucrose solution at 4°C until samples sank to the bottom. Using a SM 2000R freezing microtome (Leica Microsystems, Nussloch Gmbh, Germany) serial 30 µm thick coronal sections were cut next day that were stored in antifreeze solution (30% ethylene glycol; 25% glycerol; 0.05 M phosphate buffer; pH 7.4) at –20°C until further use.

### 2.3 Blood collection and testosterone measurement

Immediately before the start of transcardial perfusion at least 1 ml blood was collected from the opened chest cavity following incision of the right atrium. Collected blood was kept at room temperature for 30 minutes to allow clotting, then samples were centrifuged for 10 minutes at 1000 x g. Serum was removed and stored at -20°C until further analysis. Serum testosterone levels were measured using testosterone ELISA kit (R&D systems, Biotechne, KGE010) according to the manufacturer’s instructions.

### 2.4 Sample preparation and immunoblotting

Frozen testicular tissues were homogenized using an Ultra-Turrax homogenizer for approximately 15 seconds with brief pauses in buffer I (10 mM Tris-HCl, 1 mM EDTA, 0.5 mM EGTA, and 150 mM NaCl with 2x Complete Mini protease/phosphatase inhibitor cocktail in Milli-Q water) at a concentration of 50 mg/ml. Subsequently, buffer II (buffer I supplemented with 2% Triton X-100 and 0.2% SDS) was added, and homogenization was continued for an additional 5–10 seconds. The resulting homogenates were incubated on ice for 20 minutes and centrifuged at 20,000 × g for 15 minutes. The supernatant was collected for immunoblot analysis.

Protein concentrations were determined using the BCA protein assay kit (Pierce, 23225) according to the manufacturer’s instructions. Equal amounts of protein from each sample were resolved on 12% SDS–polyacrylamide gels and transferred to PVDF membranes following standard protocols. The membranes were rinsed with distilled water, stained with Ponceau S (Sigma) to verify protein transfer efficiency, washed three times with TBS-Tween (20 mM Tris, 150 mM NaCl, 0.2% Tween-20), and then blocked for 1 hour in blocking buffer (5% low-fat milk powder in TBS-Tween).

Membranes were incubated overnight at 4 °C with the following primary antibodies diluted in blocking buffer: anti-StAR (Proteintech, 12225-1-AP, AB_2115832, 1:2000), anti-CYP11A1 (Proteintech, 13363-1-AP, AB_2088552, 1:2000), anti-HSD17B3 (Proteintech, 13415-1-AP, AB_2877946, 1:2000), and anti-GAPDH (Proteintech, 10494-1-AP, AB_2263076, 1:80,000).

After three washes with TBS-Tween, the membranes were incubated with the appropriate horseradish peroxidase (HRP)-conjugated secondary antibody (anti-rabbit, 1:20,000 dilution in blocking buffer). Protein bands were visualized using enhanced chemiluminescence (ECL, Bio-Rad) and imaged with Bio-Rad ChemiDoc MP Imaging System. Images were quantified with the gel analysis tool in the FIJI software package. Band intensities were normalized to the corresponding GAPDH signal.

### 2.5 Immunohistochemistry for GnRH, AR and ERα protein expression in brain slices

After storage at –20°C, free floating brain sections were washed three times in 1× Tris buffered saline (TBS) for 10 min followed by blocking the endogenous peroxidase activity with 30% H_2_O_2_ for 15 minutes. The slices were permeabilized and blocked with 0.2% Triton-X-100 in 10% horse serum for 2 hours. Thereafter the sections were incubated with rat anti-GnRH primary antibody (generous gift from Erik Hrabovszky), rabbit anti-androgen receptor (Millipore/Sigma, 06-680, AB_310214) or rabbit anti-ERα (Santa Cruz Biotechnology, sc-542, AB_631470) diluted in TBS containing 5% horse serum and 0.02% Triton X-100 for 2 days at 4°C. Next, the slices were incubated in TBS containing biotinylated donkey anti-rat or donkey anti-rabbit IgG for 2 hours (1:300; Jackson ImmunoResearch Laboratories) following three consecutive 10 minutes wash with 1x TBS. After washing off the secondary antibodies, samples were incubated with avidin-biotin-HRP complex (1:200; Vector Elite ABC kit, Vector Laboratories) according to the manufacturer’s protocol for 2 hours. Labeling was visualized with nickel-diaminobenzidine tetrahydrochloride (DAB) using glucose oxidase resulting in a black precipitate within the labeled cells. To optimize the signal/background ratio the chemical reaction was performed under a brightfield microscopic control, and the reaction was stopped in 1x TBS at the optimal staining intensity. The stained slices were mounted onto gelatin-coated slides, air dried, and transferred into distilled water, ascending ethanol solutions (70%, 95%, and absolute for 10 min, respectively), and finally into xylene for 10 min. The samples were coverslipped using DEPEX (VWR, West Chester, PA, USA) mounting medium. All procedures, except primary antibody incubation, were carried out at room temperature.

### 2.6 Brightfield microscopy and image analysis

Brightfield microscopy was performed on a Nikon Ti-E inverted microscope equipped with a motorized x-y-z stage. Images were captured with a Hamamatsu Orca Flash 4.0 camera using NIS-element imaging software. Koehler illumination was set for the used 10x Plan Apo objective lens (N.A. 0.64). First, images of whole coronal brain slices were taken to identify the plane of the coronal brain slices. The overlapping images obtained of each whole brain slice were aligned and stitched during the acquisition creating a large 2D mosaic image.

For the analysis of GnRH or ERα-positive cells, a z-stack of brightfield images (11 slices, 4 µm steps) of the region of interest was taken using the same 10x objective. A large composite image per layer was created using the NIS-elements software. The Stack Focuser plug-in of software Fiji was applied to obtain one focused 2D image of each z-stack of large composite images. The number of GnRH neurons in the region of interest was counted manually, while GnRH immunoreactivity (GnRH-ir) was calculated using the Analyze Particles function of software Fiji after application of the Adaptive Threshold plug-in. A custom-made macro containing Otsu automatic thresholding and watershed segmentation in software Fiji was used to automatically count the number of androgen and ERα-immunoreactive cells. Imaging and image analysis were performed in a blind manner.

To determine the number and the immunoreactivity of GnRH neurons, the following planes were selected based on the Franklin and Paxinos mouse brain atlas: medial septum (MS), Plates 24-26; medial preoptic area (MPOA), Plates 28-30; lateral hypothalamus (LH), Plates 34-36. In addition to the above-mentioned brain regions the fiber density of GnRH neurons was also determined in the organum vasculosum of lamina terminalis (OVLT) and the eminentia mediana (EM). AR-positive or ERα-immunoreactive cells were counted in the RP3V (Plates 29-30) and ARC (Plates 51-53) brain regions (43). A bilateral analysis was performed on two sections in each brain region per animal to determine the GnRH-ir and the number of GnRH neurons or ERα-immunoreactive cells.

### 2.7 RNAscope *in situ* hybridization

Free-floating 30 µm thick, paraformaldehyde-fixed coronal brain slices were washed three times with TBS before mounted on Superfrost Plus Gold adhesion slides (Thermo Scientific, 630-1324, VWR). The selected transcripts were prepared and labelled according to the manufacturer’s instructions, followed by sequential amplification and detection steps (Advanced Cell Diagnostics, Newark, CA, USA.). mRNA transcripts of kisspeptin (*Kiss1)*, and androgen receptor *(AR)* or ERα (*Esr1*) were detected using a multiplex fluorescence RNAscope *in situ* hybridization assay in brain sections. To ensure specific staining of *Kiss1* transcripts, labeling of these mRNAs was performed first. *Kiss1* mRNA was labeled with Cy3 fluorophore, whereas Cy5 was used to detect *Esr1* or *AR* transcripts. Nuclei were counterstained with Hoechst 33342 and ProLong Diamond Antifade Mountant was used to cover the finished samples. Slices for negative control were labelled with 3-plex negative control probes for mouse tissue each time RNAscope labeling was performed. The slices with plate number 29–30 and 46–53 were analyzed for RP3V and ARC brain regions, respectively (43).

### 2.8 Immunofluorescent staining for tyrosine hydroxylase–positive neurons in brain slices

Brain slices with plate number 47–49 were selected for the mid ARC region. Selected slices were permeabilized and blocked with TBS containing 0.2% Triton-X-100 and 10% horse serum for 2 hours. After rinsing and 2 consecutive washes in TBS for 10 minutes the sections were incubated with guinea pig anti-tyrosine hydroxylase (Synaptic Systems, 213104, AB_2619897) at a 1:1000 dilution in TBS containing 5% horse serum and 0.02% Triton X-100 for one day at 4 °C. Following one rinsing and two 10-minute washes in TBS, slices were incubated with a donkey anti-guinea pig antibody conjugated to CF568 (Biotium, 20377) at 1:2000 dilution in TBS for 2 hours. Two rinsing steps and one 10-minute wash in TBS were followed by nuclear counterstaining with Hoechst 33342. After the final washes samples were covered with Slowfade Gold Antifade Mountant and coverslipped. All procedures, except primary antibody incubation, were carried out at room temperature.

### 2.9 Confocal imaging and image analysis of brain slices

Fluorescent RNAscope samples were imaged using a Nikon C2+ confocal laser scanning imaging system for less than one week after the samples were ready. First, a large, composite image of the entire coronal slice was created by stitching individual image tiles taken with a 10x objective (N.A. 0.64). This image was used to determine the Paxinos plate of the image slice. Next, using a Plan Apo 20x magnification objective (N.A. 0.75), z-stacks of 12-bit fluorescent images (512 × 512 pixels) were taken over the region of interest (RP3V or ARC) in a range of 5 to 15 µm below the surface of the slice with a 1 μm interslice distance, and a pinhole size less than one Airy unit. The laser power and gain of the photomultiplier tube for each channel were set during imaging slices labeled with 3-plex negative probes. All images from the same animal were captured using the same imaging parameters.

Image analysis of the obtained z-stacks was performed using Fiji software. Kisspeptin neurons were manually counted. RP3V and mid and caudal ARC brain regions were analyzed in sections with plate number 29-30, 46-49, and 50-53, respectively (43). In both regions, two sections were selected from each animal and *Kiss1* mRNA-positive cells were counted bilaterally.

### 2.10 Statistical analysis

**Figure 1:**
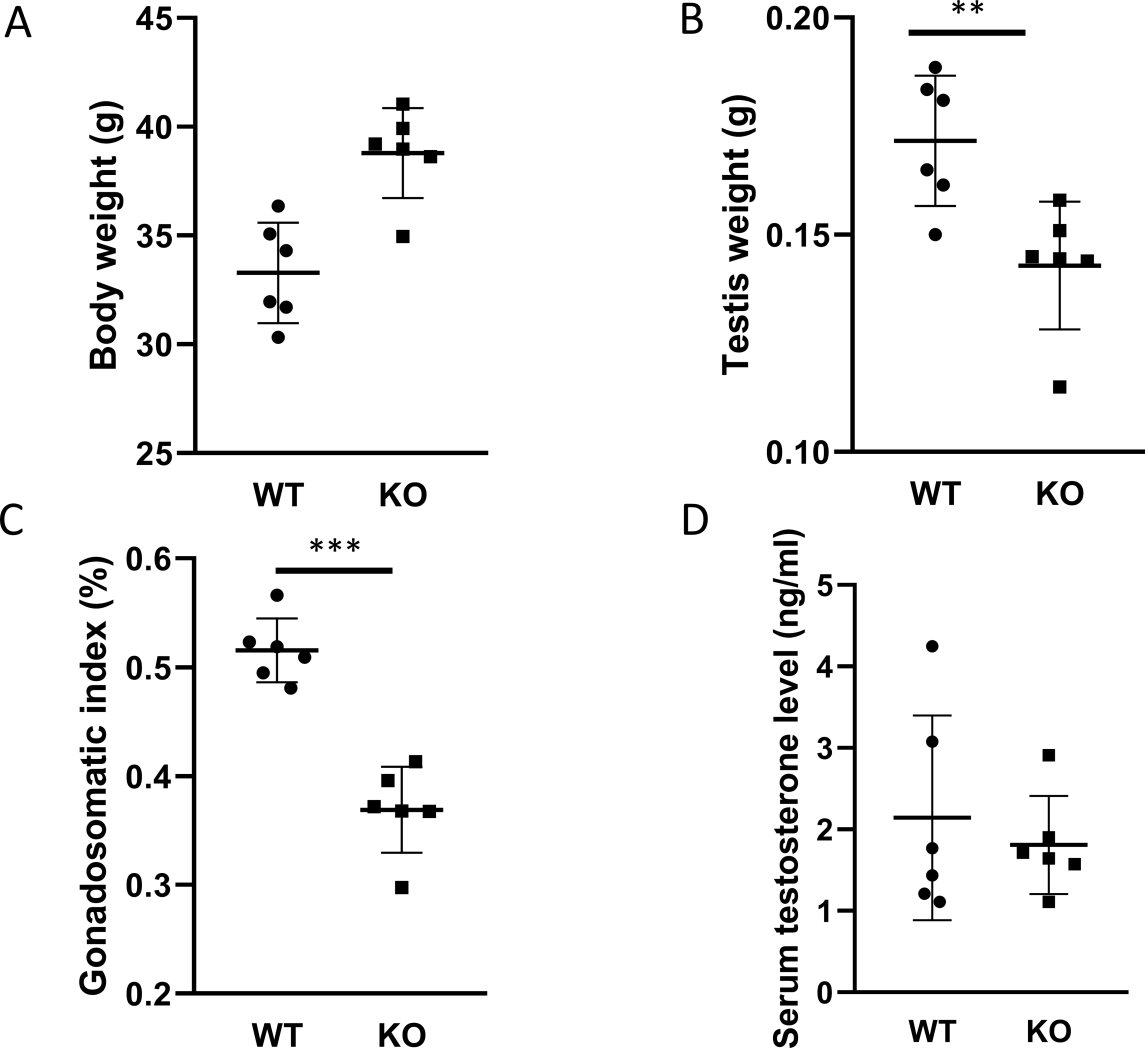
Body weight, weight of testis, and serum testosterone level. Body weight and testicular weight are shown in **(A, B)**, respectively. The calculated gonadosomatic index is presented in **(C)**. The measured serum testosterone levels in each group are shown in **(D)**. Data are presented as mean ± SD. ** p ≤ 0.01, ***p ≤ 0.001.

Statistical analysis was performed using the software GraphPad Prism. Data are displayed in dot plots. Except in figure 1, each dot represents the result obtained from one animal. Data are presented as mean ± SD or median ± range, depending on whether the data showed a normal distribution. To test for normal distribution, the Shapiro-Wilk test was applied. In case data were not normally distributed, the Mann-Whitney U test was performed. Data with a normal distribution were analyzed using an unpaired t-test. Statistical significance was set at *p* < 0.05.

## 3 Results

### 3.1 Body weight, testicular weight in WT and PACAP KO animals

Before sacrificing the animals, we measured the body weight and the weight of both testes. The animals in the PACAP KO group were significantly heavier than age-matched WT controls (p <0.01 **Figure 1A**). In contrast, the weight of testis and the gonadosomatic index of PACAP KO mice were significantly lower than that of WT animals (p <0.01 **Figures 1B**, p <0.001 **1C**).

### 3.2 Serum testosterone level and testicular steroidogenic enzyme expression

**Figure 2:**
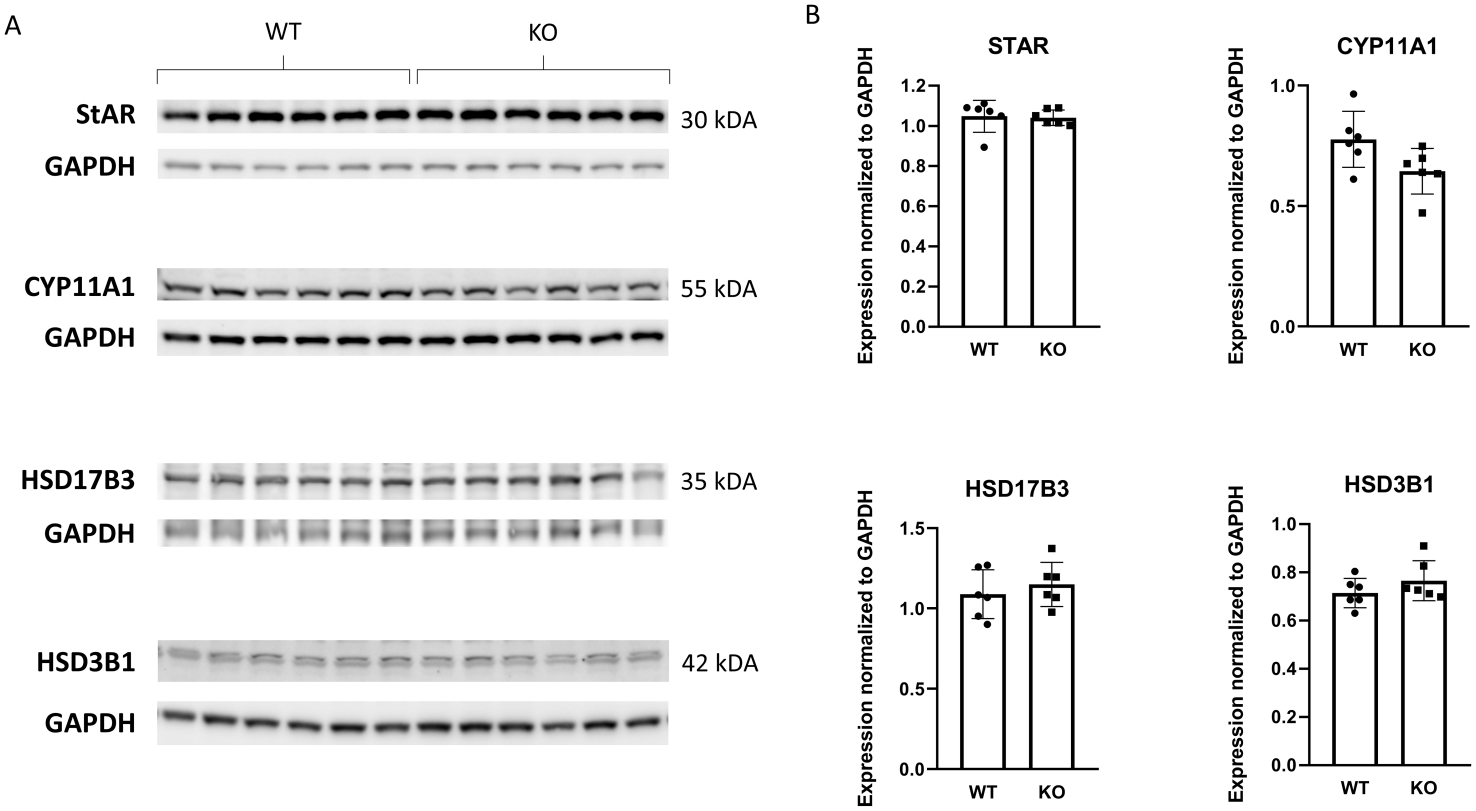
Comparison of steroidogenic enzyme expression involved in testosterone synthesis between wild-type and PACAP KO mice. In **(A)** representative images depict immunoblots of the four examined steroidogenic enzymes. Bar graphs showing the summarized data of enzyme expression normalized to GAPDH are presented in **(B)**.

Because our PACAP KO animals were on a CD1 rather than a C57Bl/6 genetic background, we examined serum testosterone levels. Unexpectedly, no significant differences were observed between the two experimental groups (**Figure 1D**). Furthermore, the testicular expression levels of four enzymes involved in testosterone synthesis in Leydig cells were also comparable (**Figure 2**).

### 3.3 Changes in GnRH-kisspeptin neural network and number of TH+ neurons in PACAP KO mice

**Figure 3:**
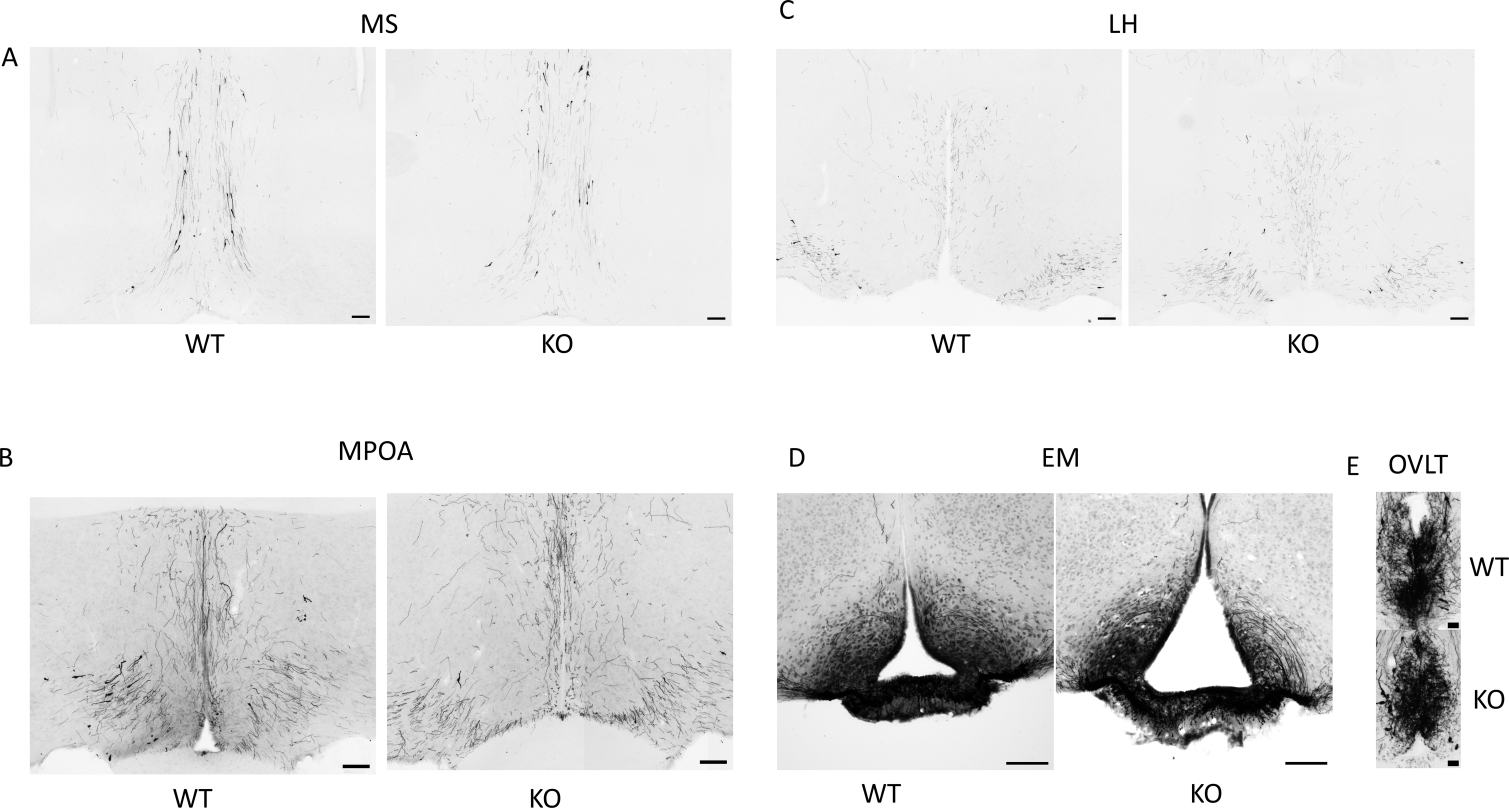
Immunolocalization of GnRH neurons in hypothalamic and septal brain regions. **(A–E)** show representative immunohistochemical images of GnRH-immunoreactive somata and fibers in the medial septum (MS), medial preoptic area (MPOA), lateral hypothalamus (LH), median eminence (EM), and the organum vasculosum of lamina terminalis (OVLT) in wild-type and PACAP KO male mice. Scale bars: 100 µm and 25 µm in **(E)**.

**Figure 4:**
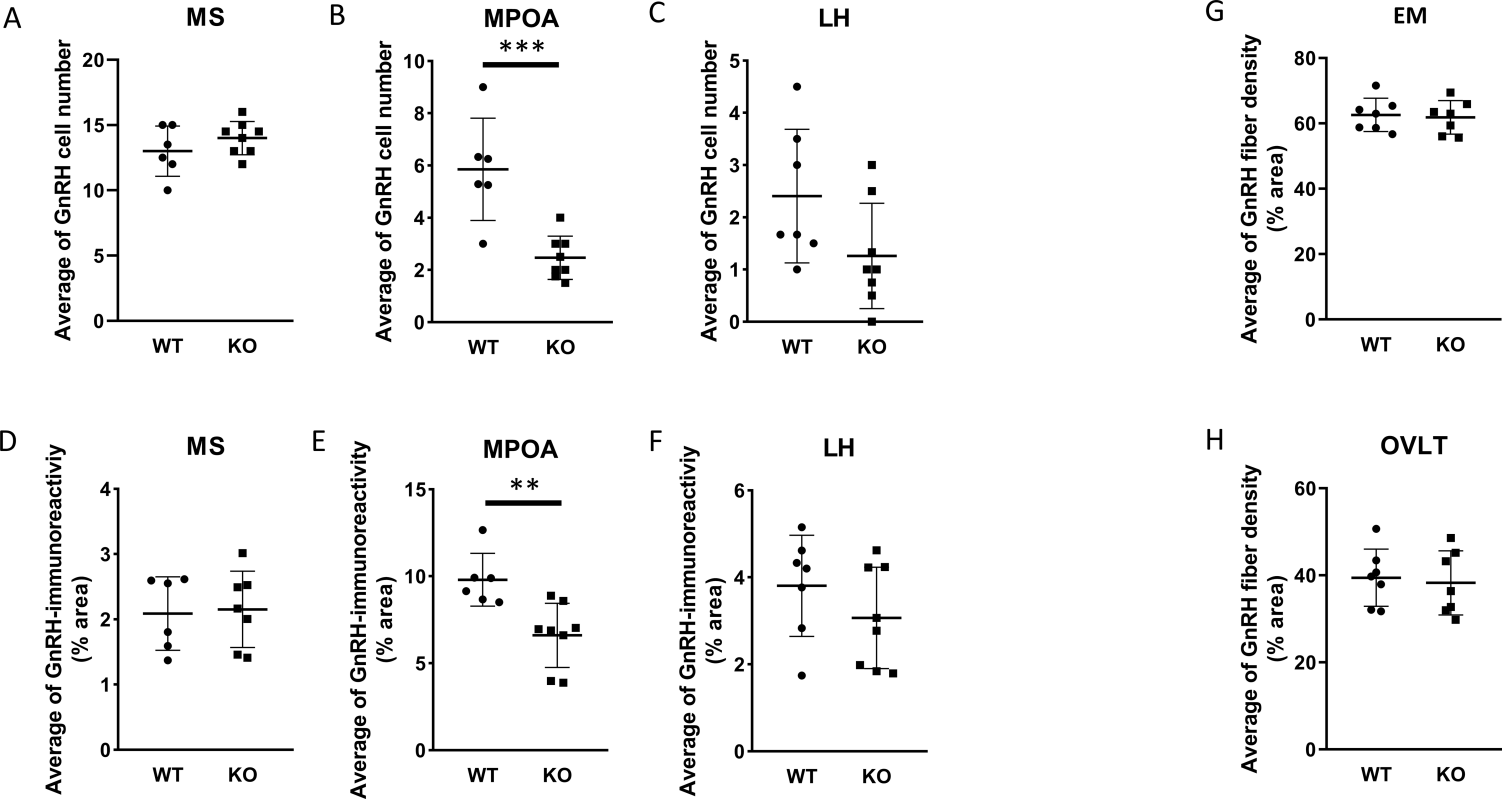
Number and arborization of GnRH neurons in various brain regions of wild-type and PACAP KO male mice. Summarized data on the number of GnRH neuron in MS, MPOA, and LH brain regions are shown in **(A-C)**, respectively. The corresponding GnRH immunoreactivity is presented in **(D-F)**, respectively. In **(G, H)**, fiber density, representing the arborization of GnRH neurons in the EM and OVLT brain areas are shown. Data are shown as mean ± SD. ** p≤ 0.01, *** p ≤0.001.

**Figure 5:**
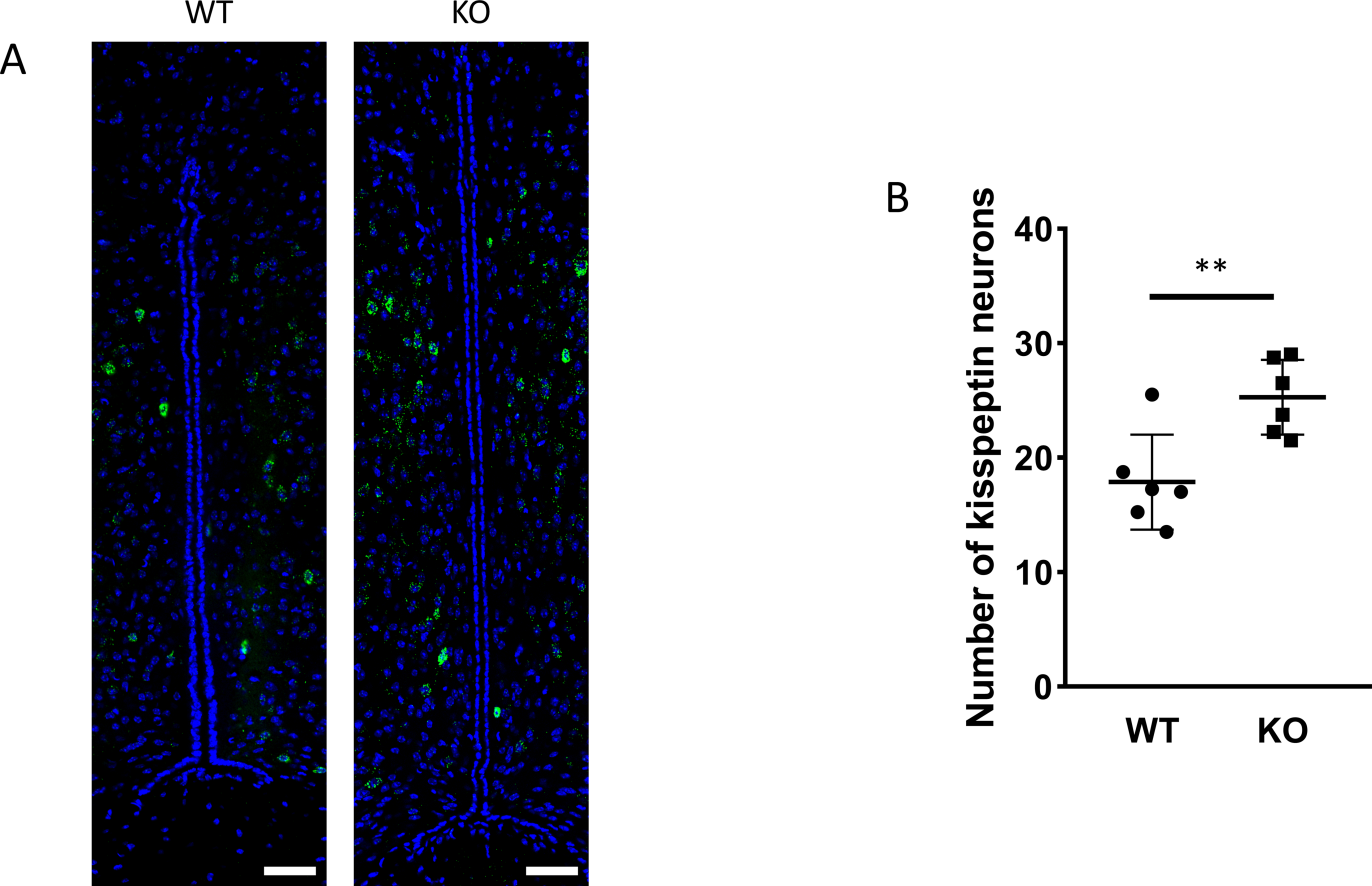
Kisspeptin neurons in the RP3V brain region. RNAscope *in situ* hybridization was used to visualize *Kiss1* mRNA expression in the RP3V hypothalamic regions of wild-type and PACAP KO animals **(A)**. The number of kisspeptin neurons in the RP3V from wild-type and PACAP KO animals are depicted in a dot plot graph **(B)**. Summarized data are shown as mean ± SD. **P ≤ 0.01, Scale bar: 50 µm.

**Figure 6:**
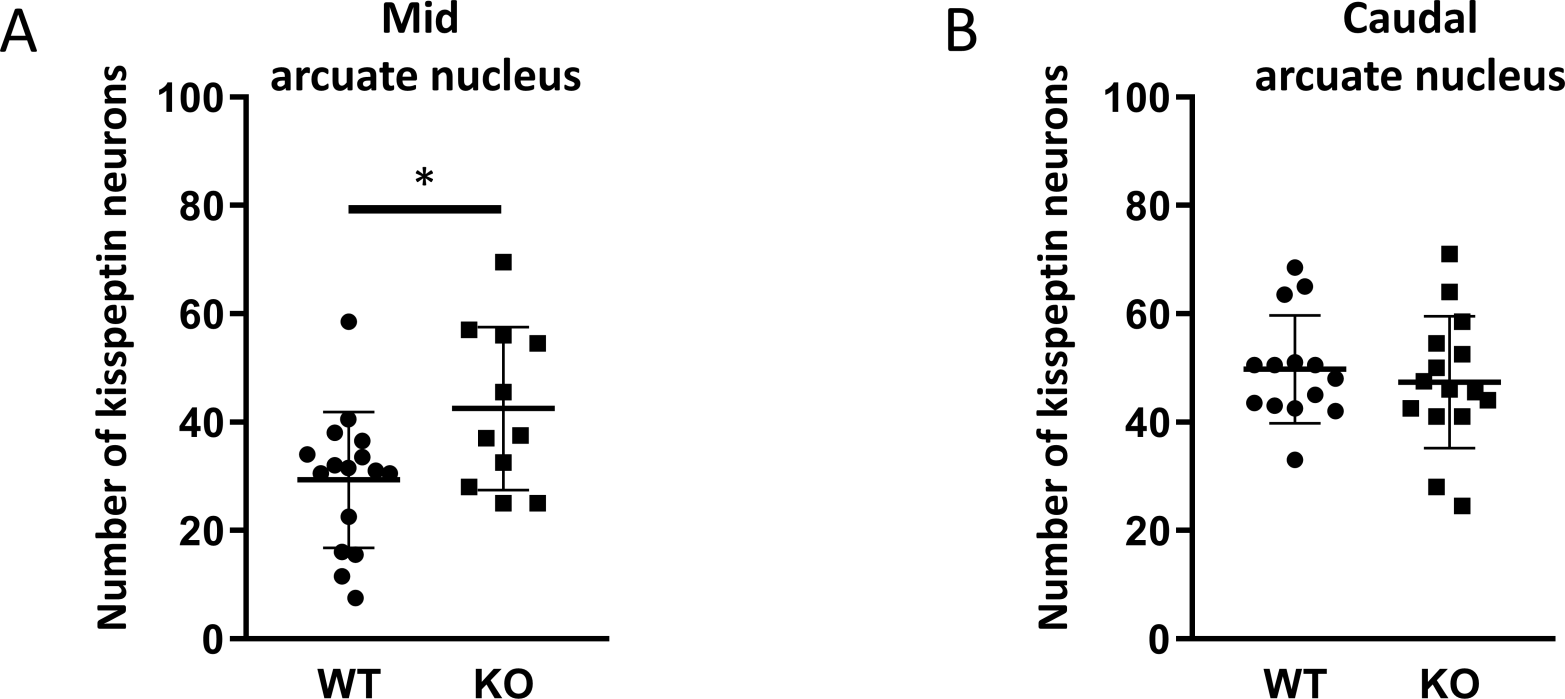
Number of kisspeptin neurons in the subregions of the arcuate nucleus in wild-type and PACAP KO male mice. Dot plots display the number of kisspeptin neurons in the mid (Paxinos 46-49, **A**) and caudal (Paxinos 50-53, **B**) arcuate nucleus. Each dot represents one brain slice. Two slides per animals were analyzed. Slices are from 10 animals per group. *p ≤ 0.05.

Because GnRH neurons play a pivotal, central role in the regulation of gonadal gametogenesis and sex steroid synthesis, first we investigated the number and immunoreactivity of these neurons in several hypothalamic regions (**Figure 3**). Immunohistochemical staining of the GnRH neurons revealed that there was a significant decrease both in the number and immunoreactivity of GnRH neurons only in the MPOA in KO animals (p <0.001 for cell number, and p <0.01 for immunoreactivity, **Figure 4**). Other examined regions (MS, LH, OVLT and ME) were not affected by the genetic ablation of PACAP (**Figure 4**). Due to the lack of a reliable antibody for detecting kisspeptin protein, we performed RNAscope *in situ* hybridization to assess kisspeptin mRNA expression in RP3V and ARC regions of the hypothalamus. We found that the number of *Kiss1*-positive neurons in the RP3V region was significantly higher in KO male mice (p <0.01, **Figure 5**). Similarly, in the mid portion of the ARC the number of *Kiss1* was higher while the caudal portion was not affected (p <0.05, **Figure 6**). To test whether the observed change in kisspeptin neurons in the middle ARC was accompanied by alterations in other types of neurons that are not part of the HPG axis but are regulated by kisspeptin neurons, we also examined the number of TH^+^ neurons in the middle ARC; however, no difference was observed between the two groups (**Suppl. 1**).

### 3.4 Expression of AR and ERα in the MPOA

**Figure 7:**
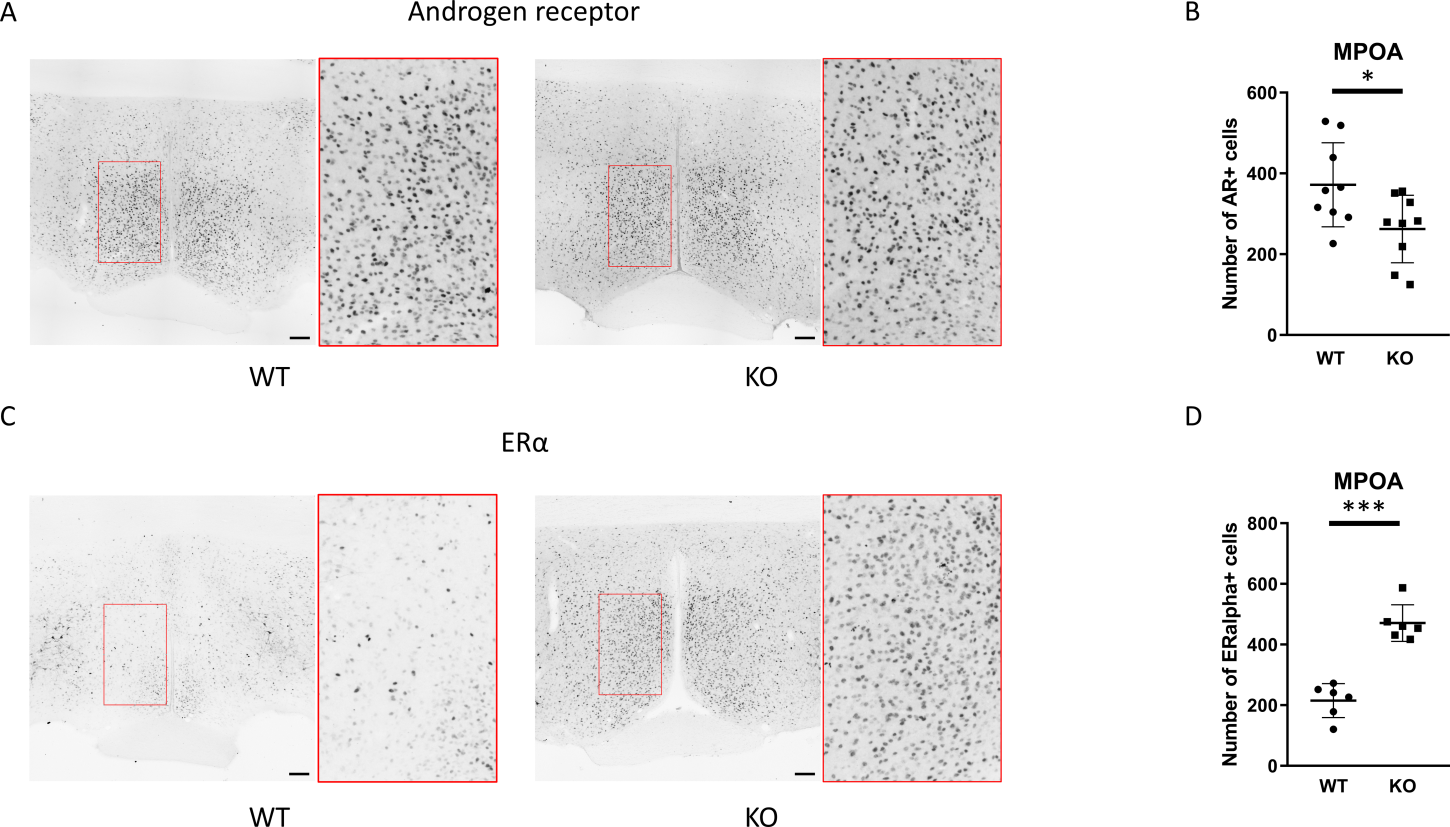
Protein expression of androgen receptor and ERα in the medial preoptic area. Representative immunohistochemical images depicting the expression of androgen receptor and ERα in the MPOA are presented in **(A, C)**, respectively. Insets display the enlarged area framed in red. Summarized data on the number of androgen receptor- or ERα-immunopositive cells in wild-type and PACAP KO mice are displayed in **(B, D)**, respectively. Data are expressed as mean ± SD. * p ≤ 0.05, *** p ≤ 0.001, Scale bar: 100 µm.

The function of the GnRH neurons in the MPOA region is regulated by a variety of neurons throughout the brain including local interneurons. A large proportion of these interneurons in the MPOA express androgen receptors and/or ERα, suggesting that they may participate in mediating the negative feedback effects of testicular steroids on GnRH neurons (44). Therefore, we examined the expression of androgen and estrogen receptors, which could serve as the site for the negative feedback of sex steroids, namely testosterone and 17β-estradiol. Immunostaining for androgen receptors revealed that the number of cells expressing ARs is lower while the number of ERα-positive cells was higher in PACAP KO mice (p <0.05 and p <0.0001, **Figure 7**).

### 3.5 Expression of *AR* and *Esr1* mRNA and protein in the RP3V and the ARC

**Figure 8:**
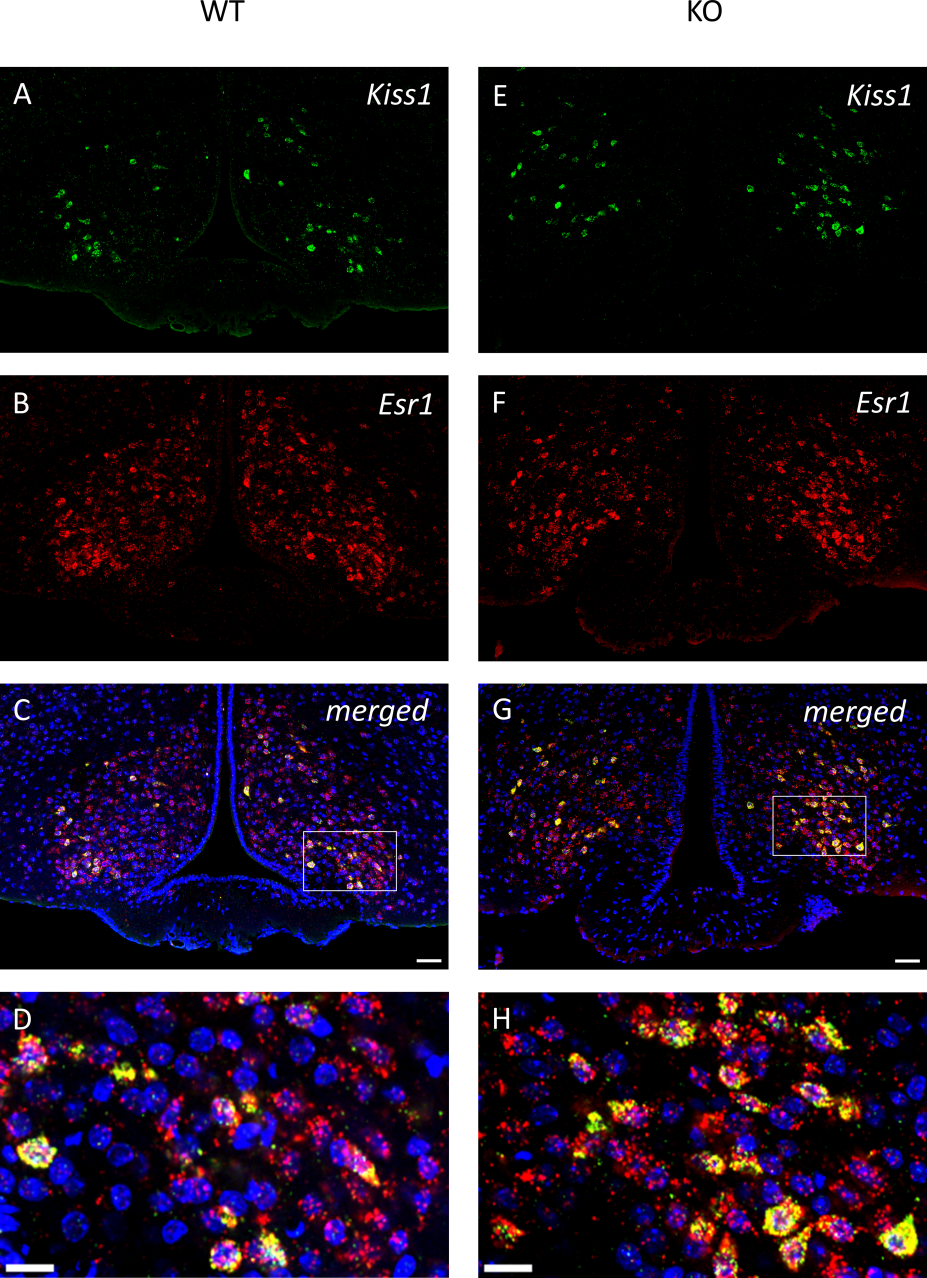
All kisspeptin neurons express *Esr1* mRNA in the middle arcuate nucleus of wild-type and PACAP KO male mice. Representative images depict the kisspeptin neurons in **(A, E)**, and *Esr1* mRNA expression in **(B, F)**, from wild-type and PACAP KO mice, respectively. Merged images are presented in **(C, G)**. Insets in **(D, H)** highlight the presence of *Esr*1 mRNA in all kisspeptin neurons. Scale bar: 50 µm, inset scale bar: 20 µm.

**Figure 9:**
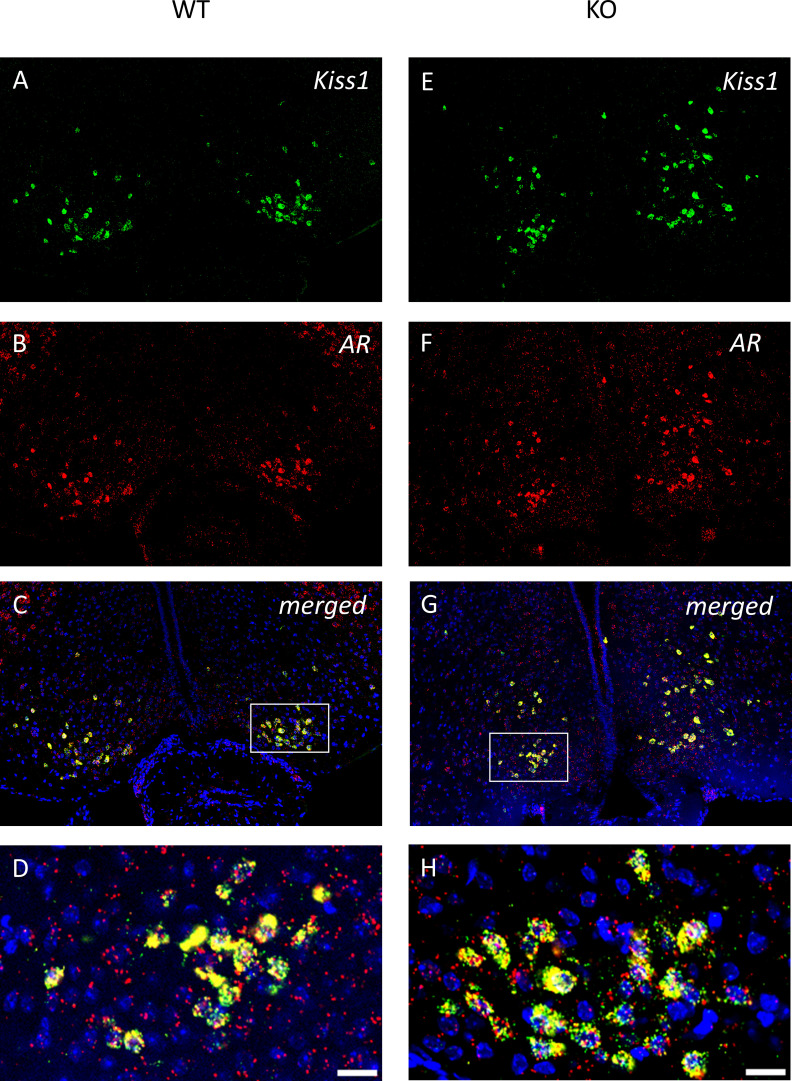
Androgen receptor mRNA is expressed in all kisspeptin neurons in the caudal ARC of wild-type and PACAP KO male mice. Kisspeptin neurons and the expression of *AR* mRNA are presented in representative fluorescent images **(A**, **B**, **E**, **F)**. Merged images presented in Panels **(C, G)**, and higher-magnification images shown in **(D, H)** visualize the expression of *AR* mRNA in all kisspeptin neurons in the ARC. Scale bar: 50 µm; inset scale bar: 20 µm.

**Figure 10:**
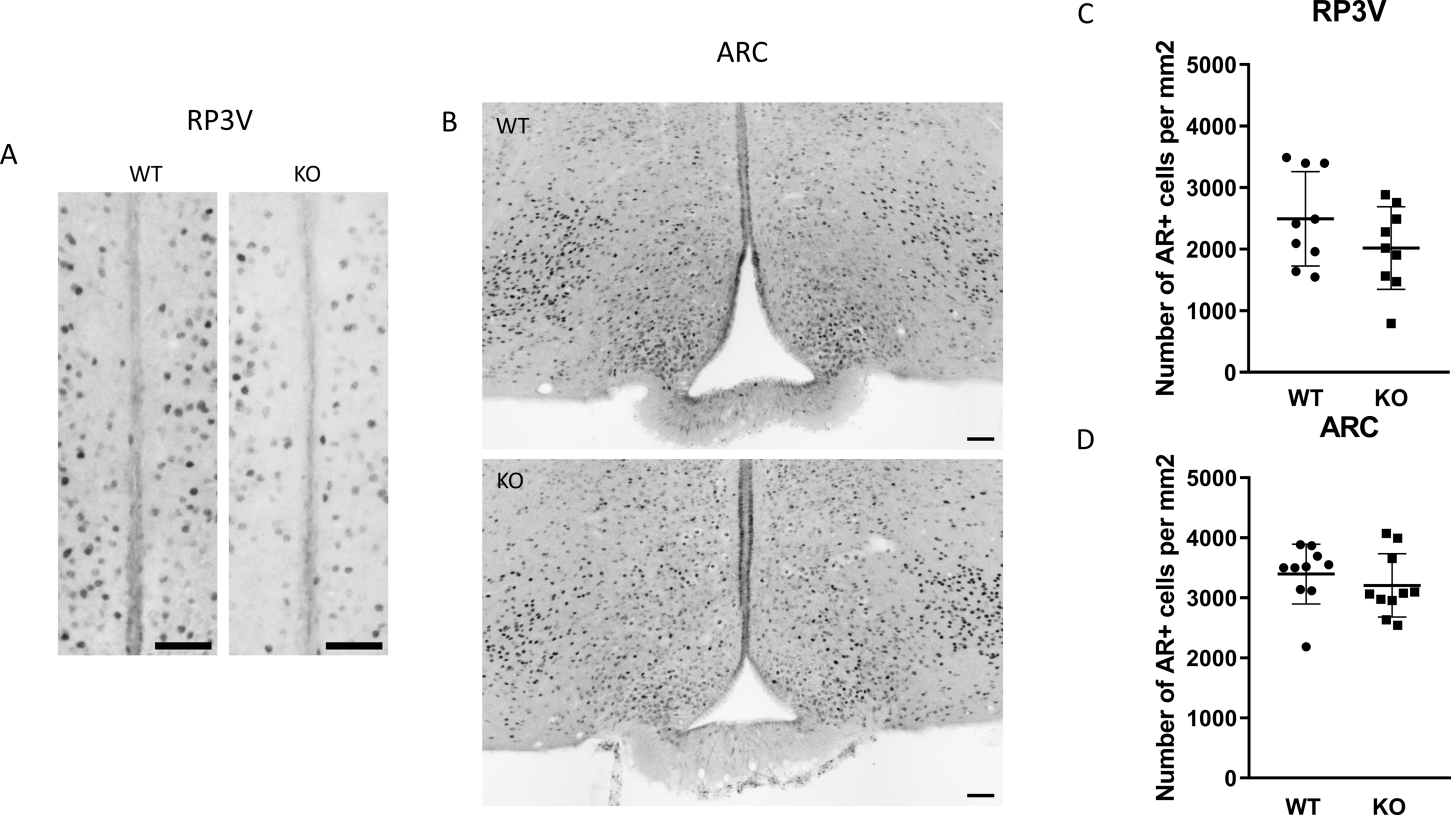
Expression of androgen receptor protein in the RP3V and the ARC. Immunohistochemical staining of androgen receptor in the RP3V region and arcuate nucleus from wild-type and KO male mouse are depicted in **(A, B)**, respectively (scale bar: 50 µm). Dot plots display summarized data from the RP3V region **(C)**, and arcuate nucleus **(D)**. Data are presented as mean ± SD.

**Figure 11:**
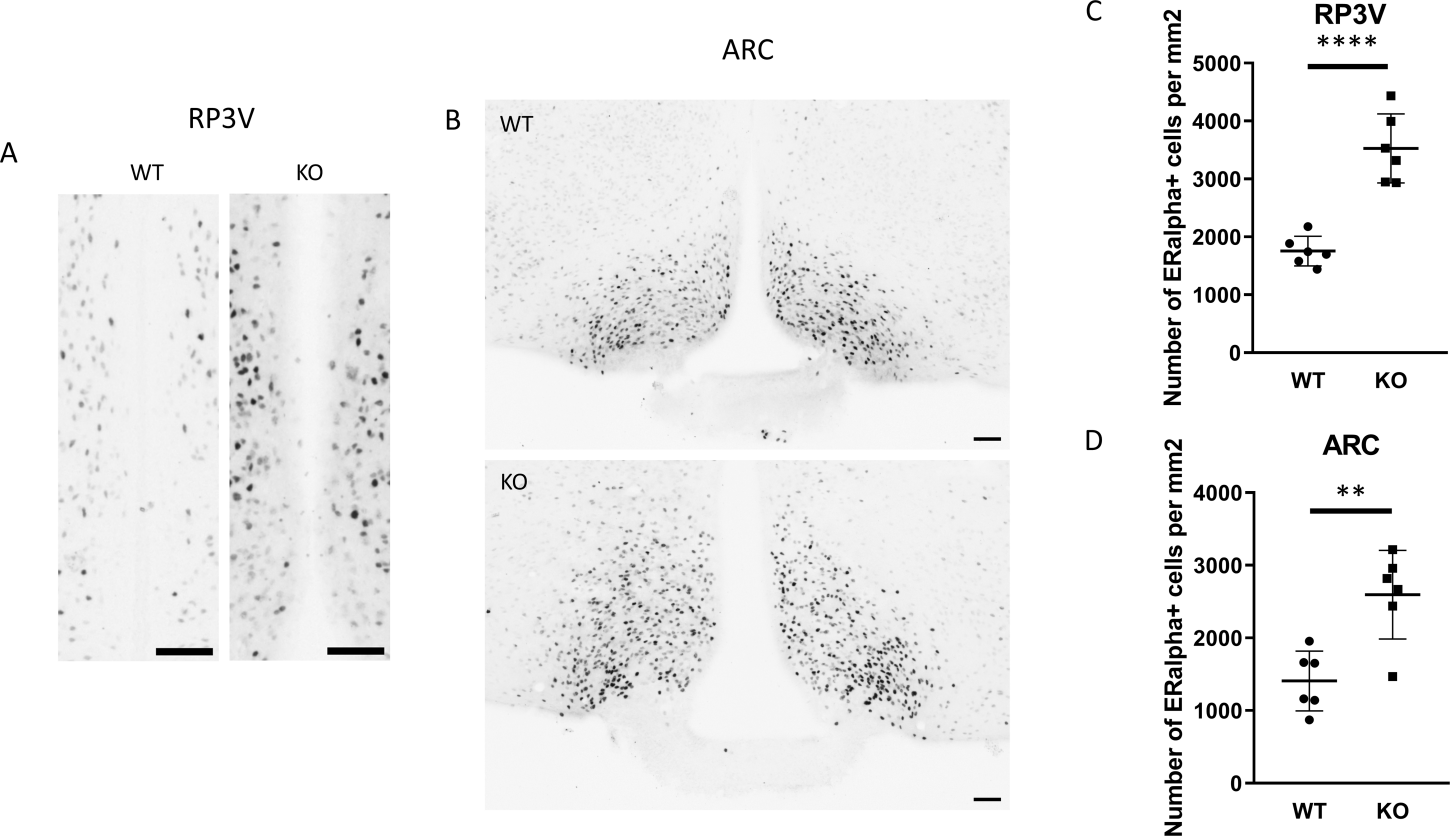
ERα-immunopositive cells in the RP3V and ARC of wild-type and PACAP KO male mice. **(A, B)** show representative images of ERα protein expression in the RP3V and ARC hypothalamic regions of wild-type and PACAP KO animals. Summarized data on ERα-positive cell counts in the RP3V and ARC are presented in **(C, D)**, respectively. ** p ≤ 0.01, **** p ≤ 0.0001.

To examine the expression of androgen and ERα receptors in *Kiss1-*positive neurons we applied RNAscope *in situ* hybridization assay to stain *AR* and *Esr1* mRNAs in the ARC. In wild-type animals *Esr1* mRNA was also present in 99.75% (796/798) of kisspeptin neuron in the ARC, while 99.67% (904/907) of kisspeptin neurons expressed *Esr1* mRNA in PACAP KO mice (**Figure 8**). In the ARC, 98.50% (786/798) and 98.88% (881/891) of kisspeptin neurons expressed *AR* mRNA in wild-type and PACAP KO animals, respectively (**Figure 9**). We also examined the number of AR and ERα-immunoreactive cells in both the RP3V and ARC regions using immunohistochemical staining. No differences in the number of AR-expressing cells were observed between the different genotypes in either the RP3V or ARC (**Figure 10**). However, the number of cells expressing ERα was significantly elevated in both regions of the PACAP KO animals (p <0.0001 and p <0.01, **Figure 11**).

## 4 Discussion

The main goal of this study was to examine the effect of PACAP on the GnRH-kisspeptin neural network within the HPG axis. When we sacrificed the animals, we found that PACAP KO animals exhibited higher body weight, but reduced testis weights compared with wild-type controls. The increased weight of PACAP KO animals is in accordance with previous reports. It is reported that genetic ablation of PACAP in the mediobasal hypothalamus, a brain region critical for energy homeostasis, induces obesity in mice (45). Furthermore, PACAP modulates hunger, satiety, and thermogenesis by acting on multiple neuronal populations within the hypothalamus, and it also exerts various peripheral metabolic effects that suppress appetite and reduce body weight (46).

The smaller testes in absence of PACAP suggest that PACAP plays a pivotal role in the physiological development of male gonads. In contrast to PACAP KO male mice on a C57/Bl6 genetic background (36), we found that serum testosterone level does not differ between wild-type and PACAP KO animals. These results are further corroborated by western blotting analyses demonstrating no difference in the levels of the steroidogenic enzymes examined between the two groups. It should be noted that most studies have reported circulating testosterone levels ranging from 1 to 10 nmol/L, whereas Lacombe et al. observed several 4-month-old wild-type males with serum testosterone concentrations between 10 and 30 nmol/L (36).

There are a variety of genetic and environmental factors that regulate reproductive functions. A large number of these elements act directly or indirectly on GnRH neurons (47). We recently reported that PACAP KO female mice with disrupted estrous cycles exhibit altered ERα expression and changes in the number of kisspeptin neurons in the ARC, while the number and immunoreactivity of GnRH neurons in the hypothalamus remained unaffected (24). In contrast, we detected a significantly lower number of GnRH neurons in the MPOA in PACAP KO males. The reduced GnRH immunoreactivity observed in the MPOA of PACAP KO animals appears to result from a lower number of GnRH neuronal somata rather than changes in fiber density. This interpretation is supported by the absence of differences in GnRH fiber density within the OVLT and ME regions. As GnRH is synthesized in the neuronal soma, a lower number of GnRH neurons would naturally lead to reduced overall GnRH production. However, since fiber density in the OVLT and ME remained unchanged, the axonal arborization of GnRH neurons must have enhanced. It should also be noted that DAB staining of GnRH neurons does not provide a quantitative measure of GnRH synthesis. Notably, the number of GnRH neurons was not different in wild-type male animals compared to our previously published data in wild-type females. Our findings regarding the sex-dependent effect of PACAP on the number of GnRH neurons in the MPOA are in accordance with the generally accepted fact that the MPOA is a sexually dimorphic region of the hypothalamus which has a crucial role in the regulation of the reproductive (parental and sexual) behavior (48, 49).

High expression of *PAC1R* mRNA, but not *VPAC1R* or *VPAC2R* mRNA, was observed in immunohistochemically identified GnRH neurons in both rats and mice using spatial transcriptomics (50). In addition, the presence of PAC1R and VPAC receptors at mRNA level was detected in immortalized GnRH cell lines (51). Although these results suggest that the observed effect of PACAP on the number of GnRH neurons could be direct, PACAP may also influence GnRH neuron number indirectly by altering the activity of neuronal populations that modulate the function of GnRH neurons. There is a plethora of neuronal inputs on GnRH neurons originating from various types of neurons such as lateral septal or hypothalamic kisspeptin neurons (4, 5, 52), GABAergic (53–56), glutamatergic (57), or neuropeptide Y (58) neurons. When we assessed how the populations of kisspeptin neurons residing in the RP3V and the ARC were affected by genetic ablation of PACAP, an increase in the number of kisspeptin neurons was detected in both the RP3V and the mid portion of the ARC. In accordance with the literature, our data also confirm that the number of kisspeptin neurons in the RP3V of males is about ~50% of that found in females, while no sex difference can be observed in the ARC. Knowing that GnRH and kisspeptin mRNA is increased by PACAP in the respective neurons, our results suggest that higher number of kisspeptin neuron is rather a consequence than a cause of the decreased number of GnRH neurons in PACAP KO animals. We speculate that the higher number of kisspeptin neurons in PACAP KO animals may compensate for the reduced GnRH neuronal population by providing enhanced stimulatory input, thereby supporting normal GnRH production and helping to preserve proper HPG axis function.

Notably, another target of kisspeptin neurons in both females and males is the TH+ dopaminergic neurons in the ARC. Tuberoinfundibular dopaminergic (TIDA) neurons, which constitute the majority of dopaminergic neurons in the ARC, receive input from kisspeptin neurons, and kisspeptin has been reported to increase prolactin secretion by inhibiting TIDA neurons in both sexes (59, 60). Although they are influenced by kisspeptin neurons, they are not considered to be part of the HPG axis. Consistent with this, despite the observed changes in the number of kisspeptin neurons in the ARC, we did not detect any differences in the number of TH+ neurons.

In males, the negative feedback of androgens and estrogens is mediated by AR and ERα, respectively (61). GnRH neurons express AR and ERα in male mice, but not in rats (50). We found that nearly all kisspeptin neurons in the ARC express both AR and ERα in the ARC. This is in line with the findings of Smith and his colleagues, who found that 87% and 64% of kisspeptin neurons expressed ERα and AR, respectively (62). The role of estrogens in male fertility and sex steroid synthesis is well-established (63). Castration of male rodents resulted in a decreased expression of ERα and AR especially in the MPOA and the ventromedial hypothalamus (VMH), which was restored by administration of testosterone (64–67). Since it was reported that PACAP affects sex steroid synthesis in testis, we investigated how the genetic ablation of PACAP changes the expression of AR and ERα in the MPOA, the RP3V, and the ARC. The most striking observation was a large, roughly 2-fold increase in the number of ERα-positive cells in the RP3V, and ARC regions, as well as in the MPOA of PACAP KO mice. Due to spatial limitations of receptor localization to individual cells in double-labeling experiments with sparse labeling, our data may suggest that kisspeptin and GnRH neurons also show increased expression of AR and ERα. Additionally, local interneurons that innervate both kisspeptin and GnRH neurons - and participate in the negative feedback loop - most likely also produce more ERα protein. In contrast, AR expression remained unchanged in the ARC and RP3V, with only a slight decrease observed in the MPOA. These data are in agreement with the fact that there was no difference in serum testosterone levels between the two groups.

Similarly to the robust elevation in ERα expression in the MPOA, it was reported that ERα immunoreactivity was increased over 200% in the same area of aromatase knockout male mice (68) showing that a reduction in the concentration of circulating estrogens would result in an increase of estrogen receptor expression in the MPOA. One plausible explanation for our data is that the lack of PACAP may disrupt in the estrogen-mediated negative feedback regulation of the HPG axis by decreasing estradiol concentration via inhibition of aromatase function in testis. The genetic ablation of PACAP can also result in lower production of FSH in the pituitary gland or direct inhibition of hypothalamic aromatase function.

In summary, we can conclude that, in our study, the genetic ablation of PACAP evokes marked changes at the hypothalamic level of the HPG axis, while the circulating testosterone level remains unaffected. The lower number of the GnRH neurons in the MPOA together with a presumably compensatory higher number of kisspeptin neurons and ERα-positive cells in the RP3V and ARC may suggest a direct hypothalamic effect on GnRH and/or kisspeptin neurons that may underlie the fertility-regulating role of PACAP in males.

## Data Availability

The raw data supporting the conclusions of this article will be made available by the authors, without undue reservation.

## Supplementary material

Supplementary Figure 1 Tyrosine hydroxylase (TH)-positive neurons in the arcuate nucleus of wild-type and PACAP KO male mice. Representative images depict TH immunostaining in the middle arcuate nucleus of wild-type and PACAP KO mice in Panels **(A)** and **(B)**, respectively. Panels **(C)** and **(D)** show summarized data on the number and immunoreactivity of TH+ neurons, respectively. Scale bar: 100 µm.
