# Supplementary figures and images for: The hypothalamic effects of PACAP on the hypothalamic-pituitary-gonadal axis in male mice
Source: Front Endocrinol (Lausanne). 2025 Nov 19;16:1677085. doi: 10.3389/fendo.2025.1677085 (PMC12673620; doi:10.3389/fendo.2025.1677085)

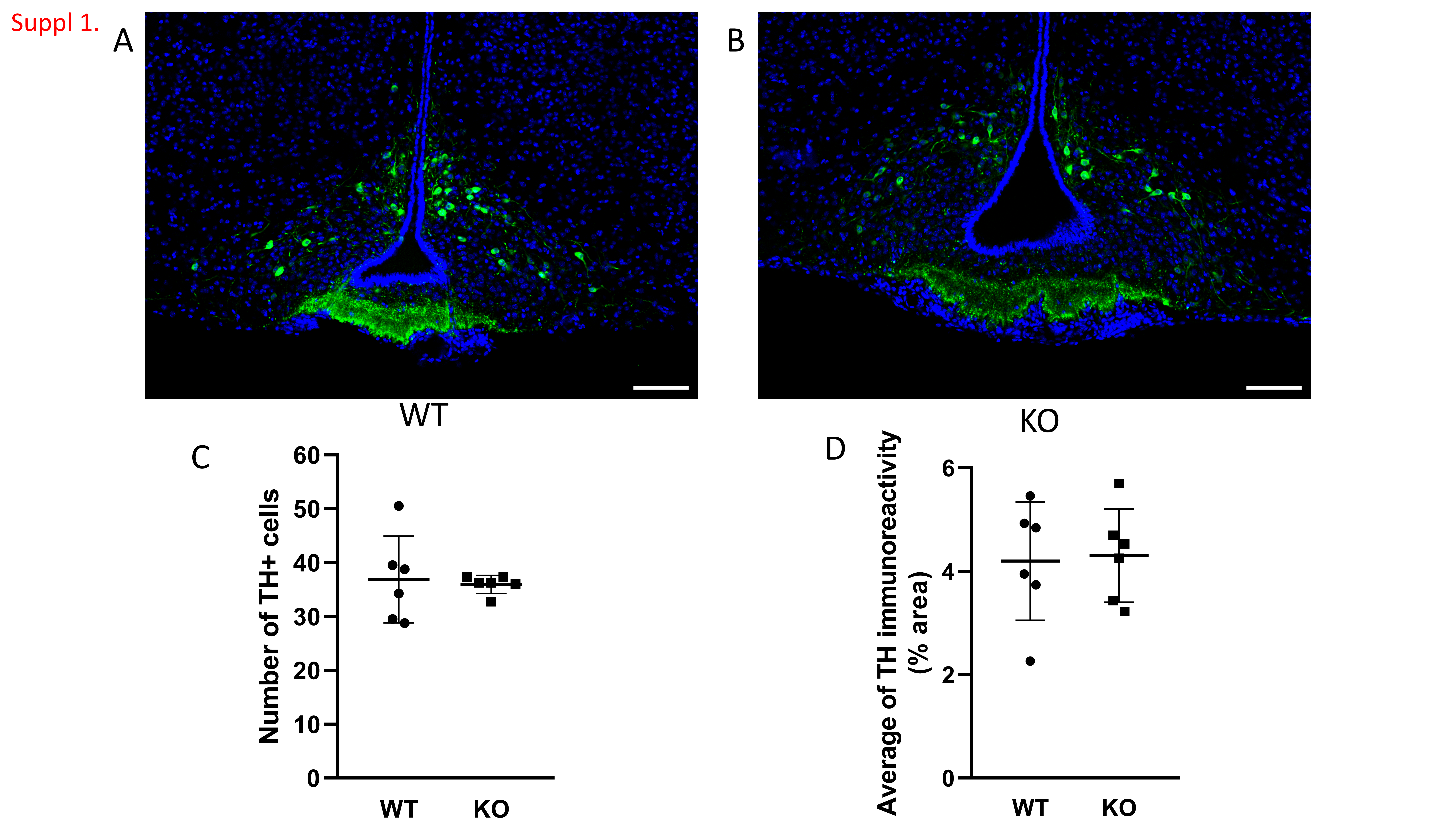

Supplement: Supplementary Figure 1 — Tyrosine hydroxylase (TH)-positive neurons in the arcuate nucleus of wild-type and PACAP KO male mice. Representative images depict TH immunostaining in the middle arcuate nucleus of wild-type and PACAP KO mice in Panels (A) and (B), respectively. Panels (C) and (D) show summarized data on the number and immunoreactivity of TH+ neurons, respectively. Scale bar: 100 µm. [file Image1.tif]
